# Genomic comparison of early-passage conditionally reprogrammed breast cancer cells to their corresponding primary tumors

**DOI:** 10.1371/journal.pone.0186190

**Published:** 2017-10-19

**Authors:** Akanksha S. Mahajan, Bruna M. Sugita, Anju N. Duttargi, Francisco Saenz, Ewa Krawczyk, Justine N. McCutcheon, Aline S. Fonseca, Bhaskar Kallakury, Paula Pohlmann, Yuriy Gusev, Luciane R. Cavalli

**Affiliations:** 1 Department of Oncology, Lombardi Comprehensive Cancer Center, Georgetown University, Washington DC, United States of America; 2 Department of Genetics, Federal University of Paraná, Curitiba, Paraná, Brazil; 3 Department of Pathology, Georgetown University, Washington DC, United States of America; 4 Division of Hematology-Oncology, MedStar Georgetown University Hospital, Lombardi Comprehensive Cancer Center, Georgetown University, Washington DC, United States of America; 5 Innovation Center for Biomedical Informatics, Lombardi Comprehensive Cancer Center, Georgetown University, Washington DC, United States of America; University of Navarra, SPAIN

## Abstract

Conditionally reprogrammed cells (CRCs) are epithelial cells that are directly isolated from patients’ specimens and propagated *in vitro* with feeder cells and a Rho kinase inhibitor. A number of these cells have been generated from biopsies of breast cancer patients, including ductal carcinoma in situ and invasive carcinomas. The characterization of their genomic signatures is essential to determine their ability to reflect the natural biology of their tumors of origin. In this study, we performed the genomic characterization of six newly established invasive breast cancer CRC cultures in comparison to the original patients’ primary breast tumors (PBT) from which they derived. The CRCs and corresponding PBTs were simultaneously profiled by genome-wide array-CGH, targeted next generation sequencing and global miRNA expression to determine their molecular similarities in the patterns of copy number alterations (CNAs), gene mutations and miRNA expression levels, respectively. The CRCs’ epithelial cells content and ploidy levels were also evaluated by flow cytometry. A similar level of CNAs was observed in the pairs of CRCs/PBTs analyzed by array-CGH, with >95% of overlap for the most frequently affected cytobands. Consistently, targeted next generation sequencing analysis showed the retention of specific somatic variants in the CRCs as present in their original PBTs. Global miRNA profiling closely clustered the CRCs with their PBTs (Pearson Correlation, ANOVA paired test, P<0.05), indicating also similarity at the miRNA expression level; the retention of tumor-specific alterations in a subset of miRNAs in the CRCs was further confirmed by qRT-PCR. These data demonstrated that the human breast cancer CRCs of this study maintained at early passages the overall copy number, gene mutations and miRNA expression patterns of their original tumors. The further characterization of these cells by other molecular and cellular phenotypes at late cell passages, are required to further expand their use as a unique and representative *ex-vivo tumor* model for basic science and translational breast cancer studies.

## Introduction

Conditionally reprogrammed cells (CRCs) are epithelial cells that grow indefinitely without the need for transduction of exogenous viral or cellular genes [1]. In this technology, epithelial cells directly isolated from either normal or malignant specimens are co-cultured with irradiated Swiss 3T3 fibroblast feeder cells (J2 cells) in the presence of the Rho protein kinase (ROCK) inhibitor (Y-27632) [2], and can be passaged long-term in tissue culture, bypassing signals for senescence. CRCs have been established from many different human [3–12] and animal [13] tumor tissues and have been used as models to study diverse cancer cellular mechanisms, including drug resistance and tumor invasion [4–7, 11]. A potential and direct clinical translation of the CRC model is the ability to assess for sensitivity a variety of chemotherapy drugs, allowing for the *in vitro* selection of the most likely effective drugs for a particular patient [3,10]. This unique possibility, offers a system where response to known drug therapies and/or novel therapeutic compounds can be directly tested on cells expanded from individual cancer patients.

A *sine qua non* requirement for the experimental use of these established CRCs is the determination of their biological representativeness in relation to their original tumor tissue, such as the maintenance of their genomic signatures after the CRC immortalization system. For most of the commercially available cancer cell lines, this comparison is not possible due to the unavailability of their original corresponding tumor tissue. Determining the genomic “fidelity” of these CRCs in relation to the tumor they derived and assessing the occurrence and effects of possible immortalization system-related effects are critical steps for the development of the system. These steps would guarantee that future molecular and/or functional downstream analysis using these CRCs can be consistently and reproducibly performed.

In this study, we performed the genomic characterization of six newly established invasive breast cancer CRC cultures in comparison to the original patients’ primary breast tumors (PBT) from which they derived. The CRCs and corresponding PBTs were simultaneously profiled by genome-wide array-CGH, targeted next generation sequencing and global miRNA expression to determine their molecular similarities in the patterns of copy number alterations (CNAs), gene mutations and miRNA expression levels, respectively. The CRCs’ epithelial cell content and ploidy levels were also evaluated by flow cytometry. A high level of overlap was observed between the CRCs and their corresponding PBTs in relation to the overall number and type of CNAs and the specific somatic variants identified. Global miRNA profiling analysis also showed a similarity in the miRNA expression levels between the CRCs and PBTs, as they clustered together with high correlation coefficients. The evaluation of the individual expression levels of specific miRNAs by real-time quantitative PCR showed no significant difference in their expression levels within each pair of CRC and PBT. These findings demonstrated that the breast cancer CRCs cultures evaluated, maintained the genome copy number, gene mutation and miRNA expression patterns of their corresponding original tumor tissue, supporting the *ex-vivo* representation of the patients’ tumor molecular signatures.

## Material and methods

### Breast cancer CRC cultures and corresponding primary tumors

**Fig 1:**
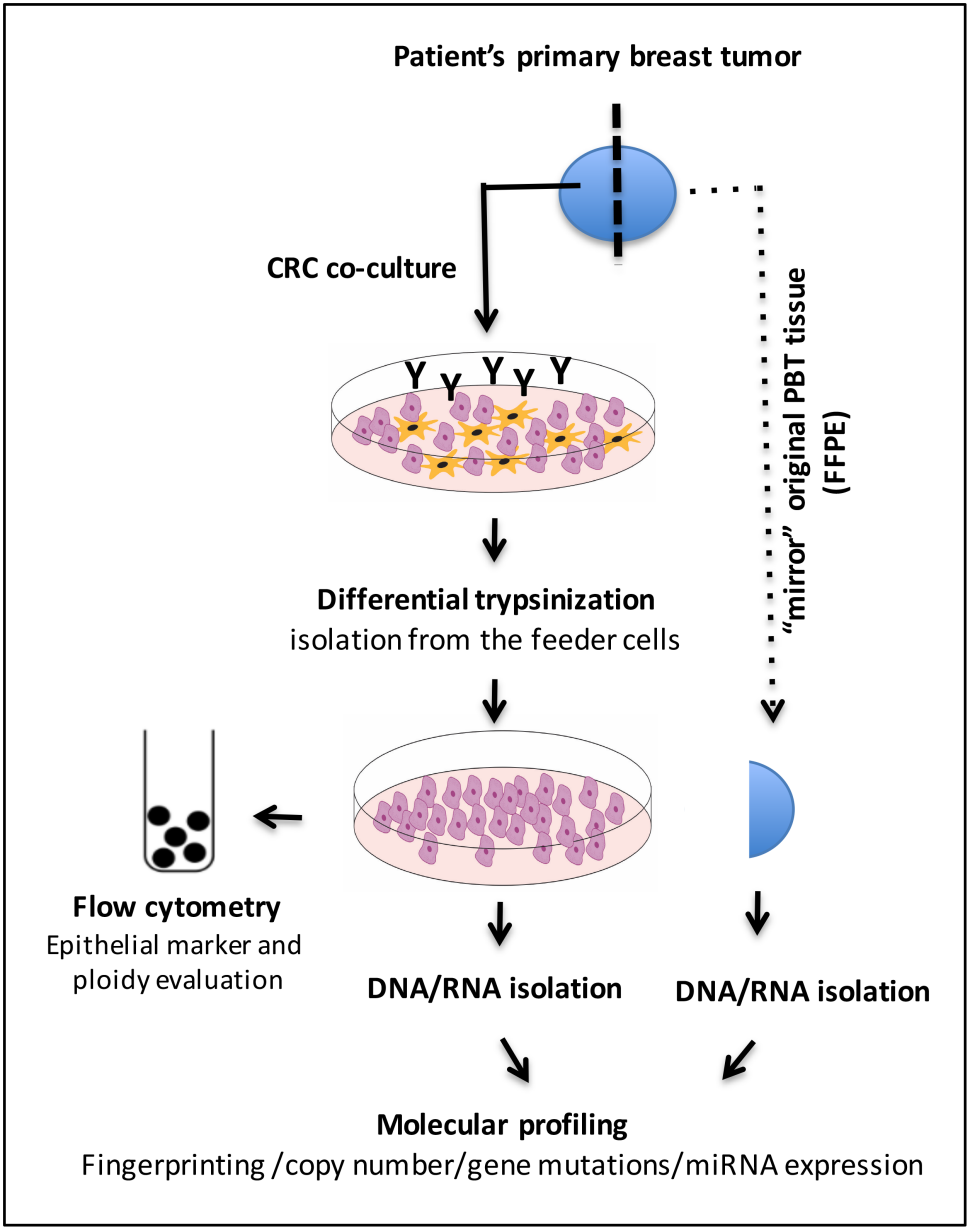
Workflow of the breast cancer CRC’s establishment using the Y-27632 compound (Y) and comparative molecular analysis of the CRCs and corresponding PBTs.

Six conditionally reprogrammed cells (CRCs) generated from fresh primary breast tumors (PBTs) were established by the Conditionally Reprogramming Cell laboratory, part of the Tissue Culture Shared Resource (TCSR) of the Lombardi Comprehensive Cancer Center (LCCC), according to an established protocol [12]. The cases were collected at MedStar Georgetown University Hospital (MGUH) at the time of the surgery, prior to any cancer treatment and under the patients’ informed consent for research and an IRB approved protocol (Histopathology Tissue Shared Resources (HTSR)- IRB#1992–048). The fresh primary tumor specimens collected were sent to the MGUH Surgical Pathology for standard histology evaluation and research assessment. An expert breast cancer pathologist (B.K.) delineated the tumor component in the resected material, for culture establishment. The mirror section of this the original resected tissue, not subjected to CRC culture, was subsequently obtained from the HTSR in formalin fixed paraffin embedded (FFPE) material. The workflow of the CRC establishment and molecular analysis is presented in Fig 1. All the experiments of this study were performed in accordance with relevant guidelines and regulations.

Relevant clinical and pathological information pertaining to the patients included: age at diagnosis, tumor size, stage and grade, and presence of lymph node metastasis (S1 Table). The average age at diagnosis of the patients was 53.33±7.87 years and the average tumor size was 4.9±4.03 cm. Most of the cases were of invasive ductal carcinoma, grades 2 and 3, except for case 1 of mixed invasive ductal and lobular carcinoma and cases 2 of invasive lobular carcinoma. In only case 1 there was no lymph node involvement. Estrogen Receptor (ER), Progesterone Receptor (PR) and Human Epidermal Growth Factor Receptor 2 (HER2) were accessed in the primary tumor tissue by immunohistochemistry (IHC) +/- FISH analysis for diagnostic purposes, following the current American Society of Clinical Oncology (ASCO)/College of American Pathology (CAP) guidelines [14,15]. Based on the “IHC subtypes”, as defined by the analysis of these 3 surrogate markers, four of the CRCs established were from hormone positive (HR+) (ER+ and/or PR+, HER2-) tumors, one from a hormone negative (HR-) (ER- and PR-) and HER2+ tumor, and one of the TNBC (ER-/PR-/HER2-) “IHC subtype”.

### CRC culture and passaging

CRCs were directly cultured using the fibroblast feeder cell system (Swiss 3T3 fibroblasts-J2 strain) according to previous protocols [1,12]. Briefly, epithelial cells were co-cultivated with irradiated 3T3 fibroblasts in F medium (3:1 (v/v) F-12 Nutrient Mixture (HAM)–Dulbecco's modified Eagle's medium (DMEM), 5% fetal bovine serum, 0.4 μg/mL hydrocortisone, 5 μg/mL insulin, 8.4 ng/mL cholera toxin, 10 ng/mL epidermal growth factor, and 24 μg/mL adenine with addition of 5–10 μmol/L Y-27632. Cells were passaged in DMEM/F12 medium containing 10 mM Y-27632 once reached 80–90% of confluence. Fibroblast feeder cells were separated from the epithelial cells by differential trypsinization. Cells were passaged until sufficient numbers were obtained for the genomic profiling and flow cytometry analysis. The assays conducted in this study were performed in cells isolated from CRCs cultured from 5 to 10 cellular passages. Cultures’ time ranged from three weeks to two months.

### DNA and RNA isolation

DNA and RNA were isolated from the CRC cultures by standard protocols once they reached 0.5x10^6^ number of cells. For their corresponding PBT, the FFPE “mirror” tissue sections containing at least 80% of tumor cells were carefully microdissected prior to DNA and RNA isolation to ensure the molecular analysis of a pure tumor cell population, as per previous protocols [16,17].

### DNA fingerprinting

Genomic authentication of the breast cancer CRCs was conducted for a subset of the CRCs in relation to their original PBTs, to ensure unequivocal donor identity. This analysis was performed by short tandem repeat (STR) profiling, as recommended by the International Cell Line Authentication Committee (ICLAC) [18] using the Promega Power Plex 16HS PCR kit (Promega, WI) and the ABI 3730 DNA Analyzer (Applied Biosystems). Allele size was performed using Soft Genetics, Gene Marker Software Version 1.85. (Softgenetics LCC, PA) (S1 Fig).

### Flow cytometry analysis

The proportion of epithelial cells within the CRC cultures was determined by flow cytometry analysis using the EpCAM fluorescence labeled antibody (APC anti-human CD326 EpCAM, BioLegend, Inc, CA). Briefly, a minimum of 0.5x10^6^ CRC cells were harvested with trypsin-EDTA and incubated with 1:1000 of antibody dilution. Non-staining cells were used as controls. The analysis was performed using the FACSAria system (BD Biosciences, NJ). A minimum of 10,000-gated cells was analyzed. Ploidy level analysis was performed in a minimum of 1x10^6^ cells. The cells were stained with propidium iodide (PI) and analyzed on FACSAria system utilizing FACSDiva and FCS Express 4 software (DeNovo Software, CA) with Peripheral Blood Lymphocyte (PBL) as an internal control. Ploidy level was calculated based on the cell cycle results. These analyses were performed at the Flow Cytometry Shared Resources (FCSR) of LCCC.

### Array-CGH analysis

DNA isolated from each of the CRC cultures and their corresponding PBTs were simultaneously profiled for copy number using an oligonucleotide array-CGH platform (SurePrint G3 Human CGH Microarray 8x60K (Agilent Technologies, CA). DNA isolated from peripheral blood from multiple normal individuals was used as reference. Digestion, labeling and hybridization were performed according to our previous protocols [16, 17]. Briefly, equal amounts of CRCs (and PBTs) and reference DNA, were enzymatically digested and directly labeled with SureTag Labeling Kit (Agilent Technologies, CA). The labeled DNA was hybridized with human Cot1-DNA (Life Technologies, CA) to the arrays, at 65°C for 40 hours. The scanned data was analyzed using the Feature Extraction (FE) software v.10.10 following importing into Agilent Cytogenomics v.2.9.2.4 software (Agilent Technologies, CA). The algorithm ADM-2 and a threshold value of 6.0 were applied with the appropriated filters to analyze the data. Gene amplifications and deletions were considered when the corresponding plotted oligo-probes presented values of log2 ≥7/6 and log2≤5/6, respectively. Duplicate experiments were performed independently for both the CRCs and corresponding PBT to assess data reproducibility.

### Next-generation sequencing

Next-generation sequencing (NGS) was performed on the Illumina MiSeq System (Illumina, Inc., CA) using the NEBNext Direct Cancer HotSpot Panel (New England BioLabs, Inc., MA). Isolated genomic DNA from CRCs and PBTs was quantified using the Quantifluor ONE dsDNA kit (Promega Corporation, WI) by following the manufacturer’s protocol. Briefly, 100 to 300 nanograms (ng) of each genomic DNA were sheared to a target size of 200 base pairs (bp) using the Covaris M220 focused-ultrasonicator (Covaris, Inc., MA). Each sheared DNA sample was enriched for DNA fragments with the NEBNext Direct Cancer HotSpot Panel, which targets 190 cancer hotspot regions in 50 genes. Each enriched DNA fragment was constructed into individual indexed libraries by following the manufacturer’s protocol. Quality and quantity of the indexed libraries were assessed using the Agilent High Sensitivity DNA kit (Agilent Technologies, CA), and were combined into a 4 nM equimolar pool. One percent of the PhiX v3 Control (Illumina) was spiked into the library pool. Paired end 2x150 bp sequencing was performed on the Illumina MiSeq using the MiSeq Reagent Micro Kit, v3 (300 cycles). Alignment to the human reference genome 19 (GRCh37, UCSC hg 19 assembly), quality and adapter trimming, and variant calling were automatically executed by the MiSeq Reporter software (version 2.6.2) on the MiSeq instrument. Annotations and filtering of all the variants were completed on the VariantStudio software (version 2.2.1) (Illumina). Variants were filtered by a mapping quality score greater than 30, read depth greater than 30, and variant frequency greater than 0.20. All synonymous and non-coding (intron) variants found outside of splicing regions were also removed. Each filtered variant was examined in the Integrative Genomics Viewer (IGV, Broad Institute) for verification and visual inspection.

### MicroRNA (miRNA) analysis

MiRNA expression analysis was performed using the Human v2 miRNA Expression Assay from NanoString nCounter Technology (NanoString Technologies, WA) as previously performed [16]. The raw data was pre-processed by NanoString’s nCounter RCC collector and the miRNAs were normalized using the geometric mean. Fold changes, represented on the log2 scale (logFC) were calculated for all differentially expressed miRNAs. Supervised hierarchical cluster (SHC) analysis was performed on miRNAs that were found to be significantly differentially expressed (P<0.05, FDR<0.05), using Pearson’s correlation coefficient and average linkage by using the Multiexperiment Viewer software (MeV 4.9.0). Gene distance matrix (GDM) analysis was also performed using MeV software to evaluate the distance of the CRCs and corresponding PBTs based on the total number of miRNAs profiled (range limits were 0.0 (lower limit) to 1 (upper limit).

### Quantitative real-time PCR

QRT-PCR was performed using TaqMan miRNA Assays (Applied Biosystems) for four individual miRNAs (miRs 125b-5p, 423-5p, 661 and 3934-5p), alleatorily selected among the 800 miRNA probes of the Nanostring platform (LifeTechnologies assays #ID000449, ID002340, ID 001606 and ID463410, respectively). CRCs and PBTs samples were normalized to the internal standard control RNA48. Each reaction was performed in triplicate, and mean value of the three-cycle threshold was used. Data was presented as means ± SE and P value≤0.05. The Student’s t-test was used for comparing the miRNA expression levels between the CRCs and the corresponding PBTs. Bonferonni correction for multiple comparisons was used and miRNAs expression was calculated by the **ΔΔ**Ct method [19].

The raw data files with miRNA expression (Nanostring) and copy number (array-CGH) data are provided as supplementary material (S1, S2 and S3 Files, respectively)

## Results

### DNA copy number analysis

**Fig 2:**
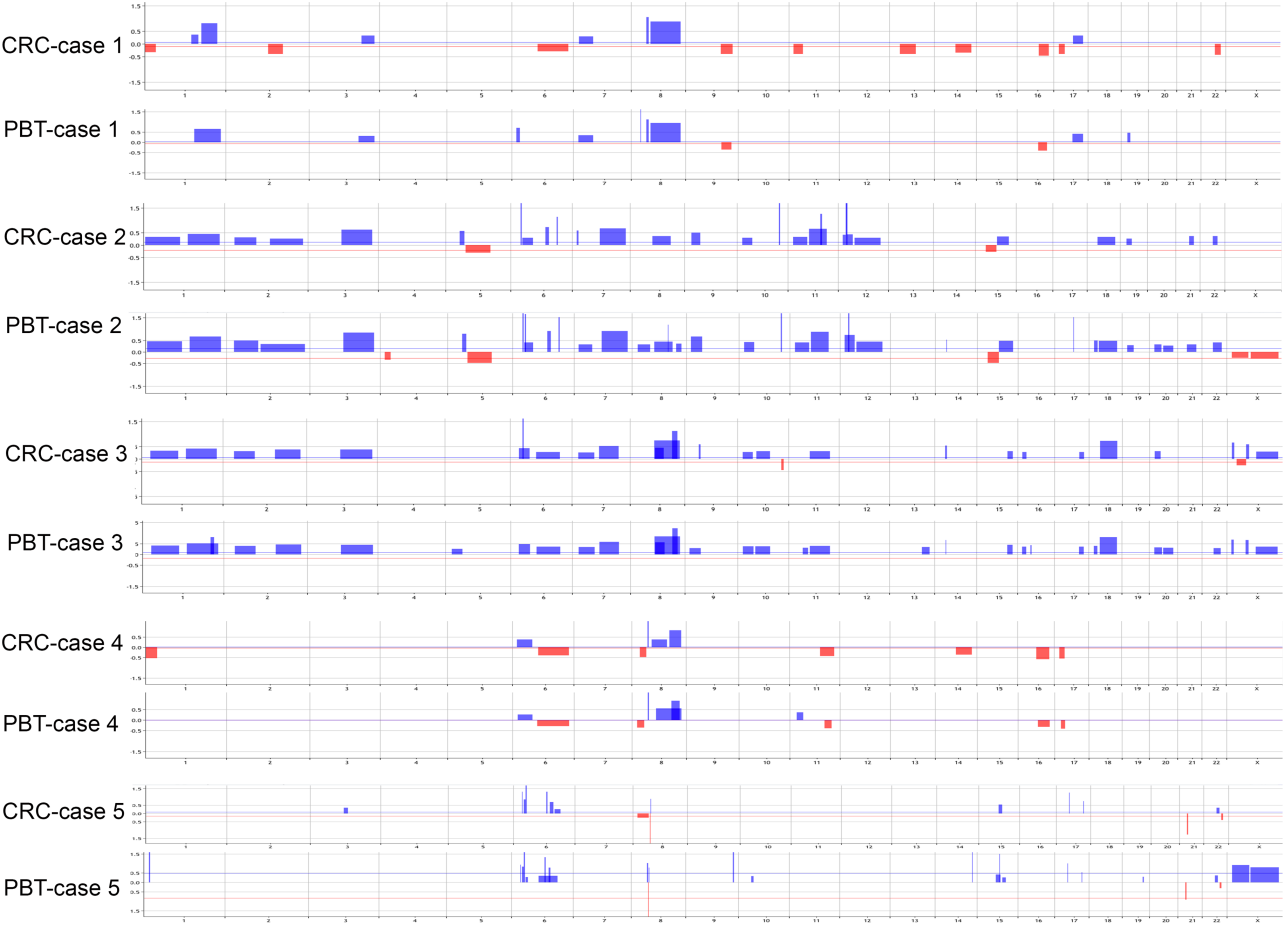
Genomic profile plots of the CRCs and corresponding original PBT of five cases analyzed. Vertical lines represent chromosome numbers and blue and red peaks cytobands with gains/amplifications and loss/deletions, respectively. Plots obtained from Agilent Cytogenomics v.2.9.2.4, using the algorithm ADM2 and the threshold value of 6.0.

Genome-wide copy number analysis was performed by array-CGH in all the six established breast cancer CRCs and their corresponding PBTs analyzed. Copy number alterations (CNAs) were observed in all the CRCs profiled. The average number of CNAs observed in these cells was 25.50±14.79, which was not significantly different from the average number of the CNAs observed in the PBT group (29.33±18.01) (unpaired t test; t = 0.696, P>0.05) (Fig 2, Table 1).

**Table 1:** Total number of copy number alterations (CNAs) observed between the CRCs and corresponding PBTs of each pair analyzed and their respective common cytobands and range of overlap (in bold CNAs with overlap >95%).

Case # a	Total # CNAs	Common Cytobands with CNAs	Range of overlap
Case 1	CRC = 17 PBT = 11	+1q23.3-q25.3, +1q31.3-q42.2, +3q25.31-q29, +7p22.1-p12.2, **+8p12-p11.21, +8q11.21-q24.3,** -9q22.33-q34.13, -16q13-q24.1, **+17q21.33-q25.3**	82–100%
Case 2	CRC = 29 PBT = 40	**+1p36.23-p12, +1q21.1-q44,** +2p24.2-p11.2, +2q21.2-q37.3, **+3q11.1-q29, +5p13.3-p12, -5q11.1-q23.3, ++6p22.3,** +6p22.3-p12.2, +6q16.1-q21, **+6q23.3, +7q11.23-q36.3, +8q12.1-q23.3**, +9p24.1-p13.3, **+10p15.2-p11.21, ++10q26.12-q26.13, +11p15.4-p11.12, +11q11-q23.2, ++12p13.2, +12q12-q24.31,** -**15q11.1-q21.3**, +15q21.3-q26.2, **+18q11.2-q23**, +19p13.2-p12	74–100%
Case 3	CRC = 28 PBT = 33	**+1p35.3-p12, +1q21.1-q44**, **+2p24.1-p11.2, +2q24.1-q37.3**, **+3q11.1-q29,** +6p23-p12.3, **+6q13-q24.3**, **+7p22.1-p11.2, +7q11.23-q33, +8q12.3-q24.3**, +8q13.1-q22.1, **+8q24.12-q24.23**, +10p15.2-p11.21, **+10q11.22-q23.31, +11q11-q23.3,** +14q11.2-q12, **+15q25.2-q26.3, +16p13.3-p13.12, +17q24.2-q25.3, +18q11.2-q23,** +20p13-p11.22, **-Xp22.33-p22.31, +Xp11.23-p11.21, +Xq21.1-q28**	72–100%
Case 4	CRC = 11 PBT = 10	**+6p25.3-p12.3, ++8p12-p11.21**, +8q11.21-q22.2, +8q23.1-q24.3,**+11q14.2-q25,** -16q12.1-q24.3	92–100%
Case 5	CRC = 16 PBT = 25	+6p23-p22.3, **+6p22.3-p22.2**, +6p22.2, 6q16.1, —8p11.22, +8p11,21-p11.1, +15q21.2-q22.2, +17q11.2, +17q25.1, -21p11.2, **+22q12.1-q12.3, -22q13.2–13.32**	76–100%
Case 6	CRC = 52 PBT = 57	**+1p32.3-p21.1, +1q21.1-q44**, +1q32.1-q44, -2p25.3-p24.1, -**2q13-q23.3, +2q23.3-q32.2, -3p24.1-p12.3, -3q28-q29, -4p16.3-p13, +4q12-q21.21, -4q21.21-q28.3,** -5p14.1-p12, **-5q11.1-q35.3, -5q11.1-q13.2, +6p25.3-p22.1, -6q23.3-q27, -7p22.3-p11.2, +7p21.2-p15.3, —7q11.21-q22.1,** +7q34-q36.3, **+8q12.1-q24.3, +8q22.1-q21.21, +8q24.21-q24.3, +9p24.3-p22.3, -10q23.32-q24.1, +11q14.1-q22.1, +11q22.3-q25, +12p13.33-p11.21, -13q14.2-q22.1, +13q32.1-q34, -14q11.2-q32.33, -15q11.1-q26.3,** -15q26.2-q26.3, **-16p13.3-p11.1, —16p11.2,** -16q12.1-q24.2, -17p13.3-p11.2, **+17q24.1-q25.3,** +18q11.1-q12.1, **-18q21.1-q23, -19p13.3-p13.2, +19p13.2-p12,** -19q13.32-q13.43, **-20p13-p11.23, +20q11.21- q13.33**, -22q11.1-q13.31	82–100%

The comparison of the array-CGH profile of each CRC with its corresponding PBT showed a similar pattern of CNAs (Fig 2). The affected cytobands and the type of CNAs observed (gain/amplifications and/or loss/deletions) between each paired CRC/PBT presented 72–100% of overlapping levels, as reported by the common interval analysis (Agilent Cytogenomics v.2.9.2.4 software). For the cytobands most commonly affected by CNAs (based on the highest P values of CNAs), such as gains at 1p36-p12, 1q21-q44, 6p25-p12, 6q13-q24, 7q11-q36, 8q12-q24, 11q11-q23 and 17q21-q25, more than 95% of overlap was observed between each CRC and corresponding PBT (Table 1).

**Fig 3:**
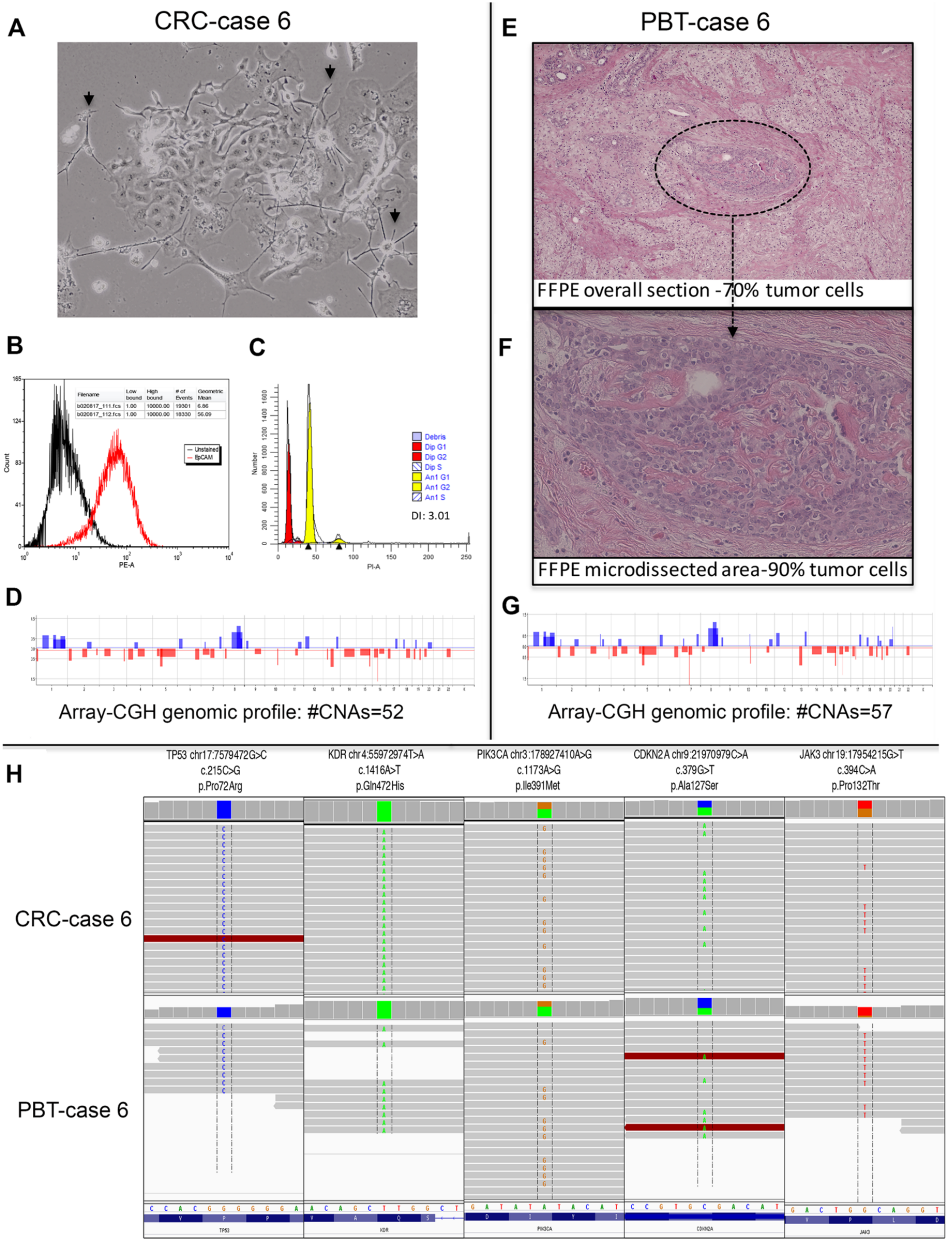
Primary breast tumor (PBT) and corresponding CRC of case 6, representative of the TNBC subtype. **A**: Phase contrast image of CRC co-culture, showing the feeder cells (short arrows) and an epithelial cell colony (cobblestone cell morphology) (40x); **B**: Flow cytometry histogram showing CRC cells stained for PE/CD326 (EpCAM) (red peaks); unlabeled control (black peaks). Number of gated cells >10,000; **C**: Ploidy analysis showing a DNA index of 3.01(G1 aneuploidy yellow peak) in relation to the diploid control (Peripheral Blood Lymphocyte (PBL)—G1 diploid red peak); **E**: FFPE tissue section (40x) and **F**: tumor area microdissected for the molecular analysis (400x); **D** and **G**: Genomic profile plots of the PBT and corresponding CRC, respectively; **H**. Next generation sequencing analysis of CRCs and corresponding PBTs showing the retention of specific somatic variants on the *TP53*, *KDR*, *PIK3CA*, *CDKN2* and *JAK3* genes in the CRCs.

**Fig 4:**
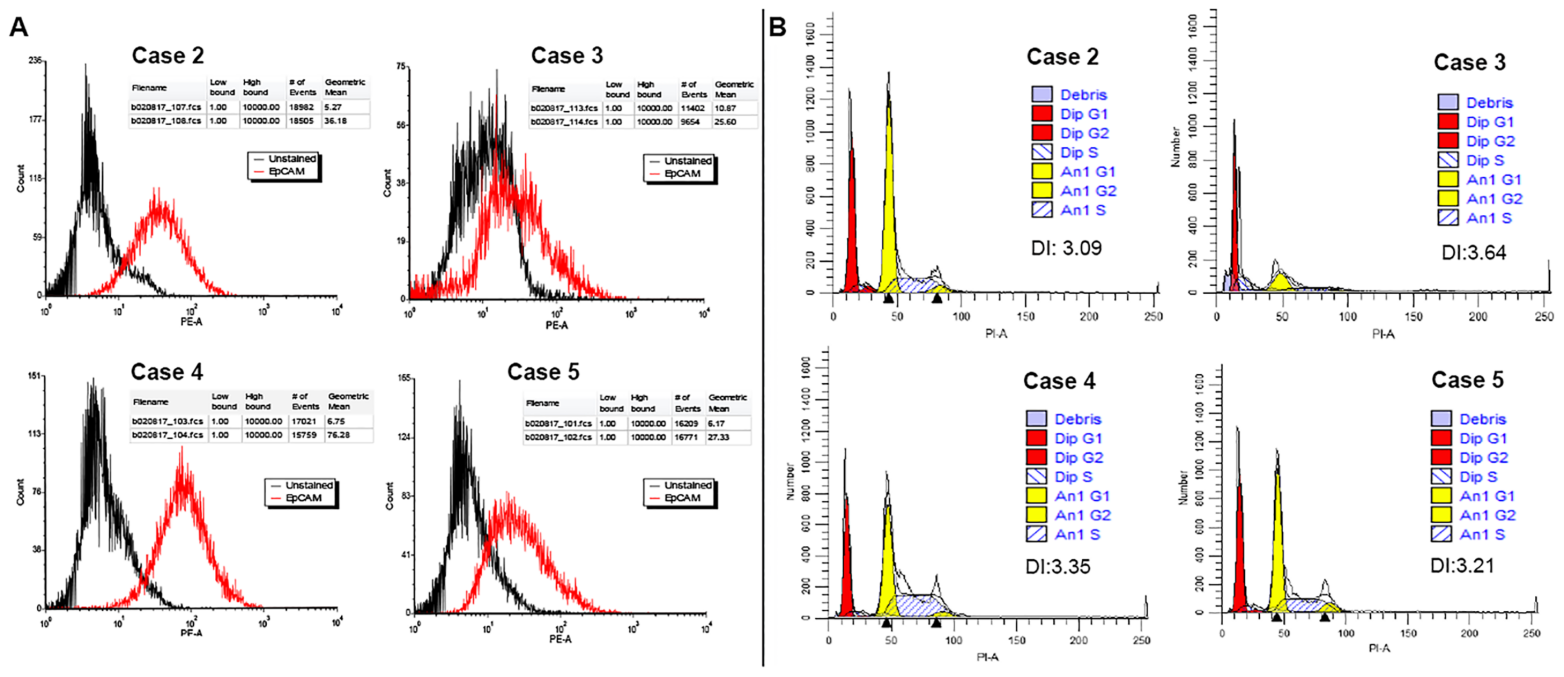
**A.** Flow cytometry histogram of CRCs from cases 2–5, showing CRC cells stained for PE/CD326 (EpCAM) (red peaks); unlabeled control (black peaks). Number of gated cells >10,000. **B**. Ploidy plots and corresponding DNA index of the CRCs (G1 aneuploidy yellow peak) in relation to the diploid control (Peripheral Blood Lymphocyte (PBL)—G1 diploid red peak) (FACSAria system).

The CRC of case 6 (Fig 3A), originated from a patient with triple negative breast cancer (TNBC), similar to its corresponding PBT (Fig 3E and 3F) presented the highest number of CNAs (total of 52 and 57, respectively) (Fig 3D and 3G). This case presented a high percentage of epithelial cells (56.1% of EpCAM gated cells) and a DNA index level of 3.01 in relation to the diploid control as verified by flow cytometry analysis (Fig 3B and 3C). In the other CRCs, the content of epithelial cells and DNA index levels ranged from 25.6% (case 3) to 76.3% (case 4) and 3.09 (case 2) to 3.64 (case 3) (Fig 4A and 4B)

### Targeted next-generation sequencing

Targeted next-generation sequencing was performed on three pairs of CRCs and corresponding PBTs (cases 2, 4 and 6) and in one unpaired CRC line (case 3) using the MiSeq platform and the NEBNext Direct Cancer Hotspot Panel. The MiSeq run yielded 1.15 gigabases (Gbp) of data with 96% >Q30 (1.11 Gbp), and 8.1 million reads. One sample (PBT of case 6) had a low library concentration and did not produce as many reads (14,480 reads) compared to the other samples. Although the read depth was low in this sample, the variants called presented a >35% allelic frequency and were cleanly visualized in the Integrative Genomics Viewer (IGV) (Broad Institute, MA) (Fig 3H). Common somatic variants to the CRCs and PBTs were found affecting the Cyclin Dependent Kinase Inhibitor 2A (*CDKN2A)*, FMS Related Tyrosine Kinase (*FLT3)*, Janus Kinase *3 (JAK3)*, Kinase Insert Domain Receptor (*KDR*, *alias VEGFR2)*, Phosphatidulinositol- 4,5-Bisphosphate 3-Kinase Catalytic Subunit Alpha (*PIK3CA)*, and Tumor Protein P53 *(TP53)* genes (S2 Table). Except for the *FLT3* gene, which presented a splice region mutation type, the other genes were affected by missense mutations. For case 3, where only the CRC culture was sequenced, missense somatic mutations were present in the *CDKN2A*, *KDR*, *KIT* Proto-Oncogene, Receptor Tyrosine Kinase (KIT), *JAK3*, MET Proto-Oncogene, Receptor Tyrosine Kinase *(MET*) and *TP53* genes.

### MiRNA profiling analysis

**Fig 5:**
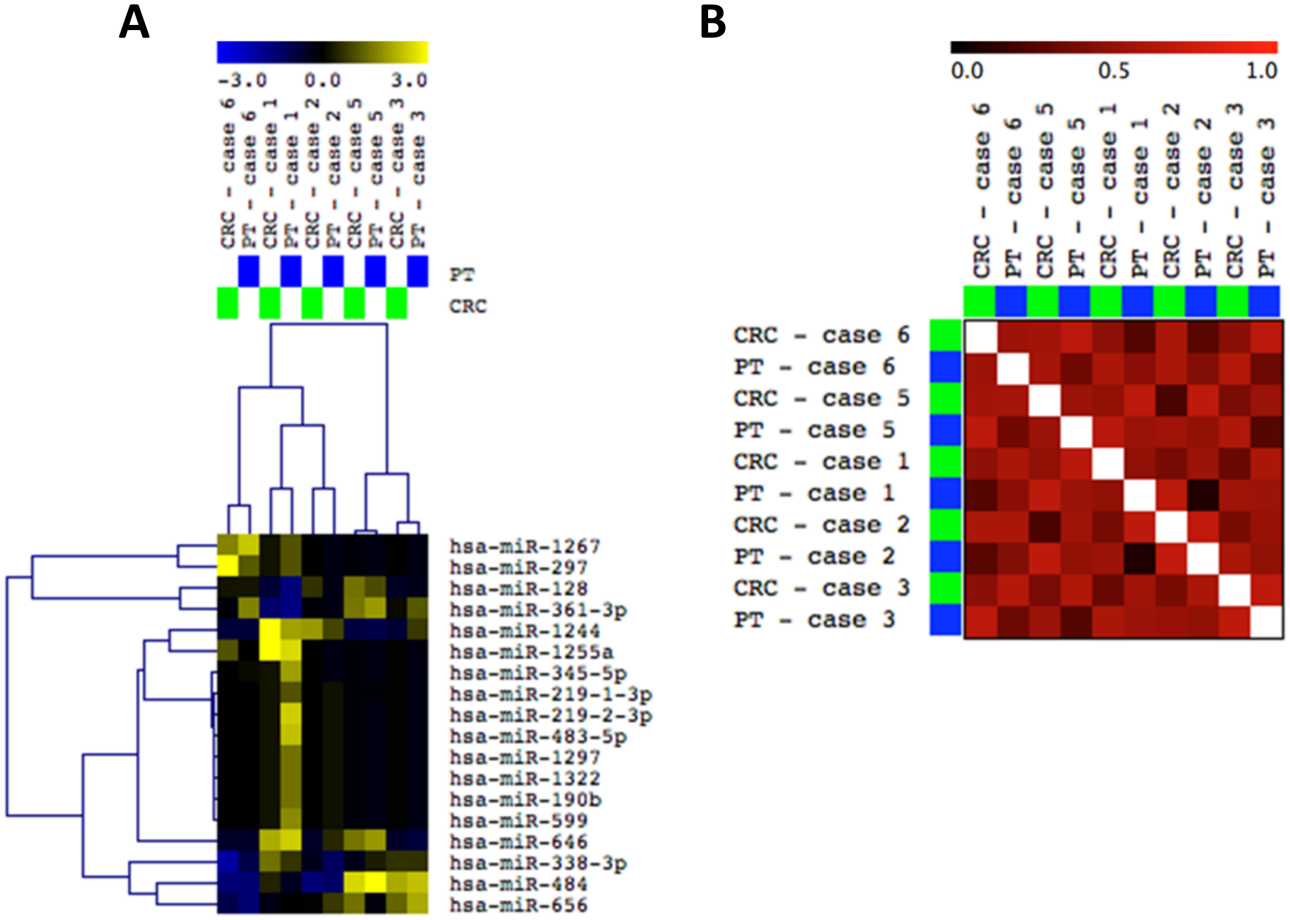
Global miRNA profiling of five pairs of CRCs and corresponding PBTs. **A:** Supervised Hierarchical Clustering (SHC) analysis (Pearson Correlation, Anova P<0.05) showing close clustering for most of the paired cases based on 18 miRNAs differentially expressed (miRNAs up-and down-regulated in yellow and blue colors, respectively). **B:** Gene Distance Matrix (GDM) correlation analysis, respectively (MeV 4.9.0).

Global MiRNA profiling was performed in five breast cancer CRC cultures and corresponding PBTs (except case 4, where only the CRC line presented sufficient quantity and quality of RNA for this analysis). The comparison of the miRNA profiling (of a total of 800 miRNA probes distributed throughout the genome) of the CRCs and their corresponding PBT, as for the array-CGH analysis, also showed an overall concordance of the expression levels of the miRNAs analyzed. Eighteen miRNAs differentially expressed among these cases (P<0.05), clustered the CRCs with their corresponding PBTs (Supervised Hierarchical Clustering (SHC), Pearson Correlation Analysis, Anova P<0.05) (Fig 5A). Gene Distance Matrix (GDM) analysis based on these differentially expressed miRNAs showed a high correlation score (>0.6) for most of the pairs of CRCs and PBTs analyzed (Fig 5B).

**Fig 6:**
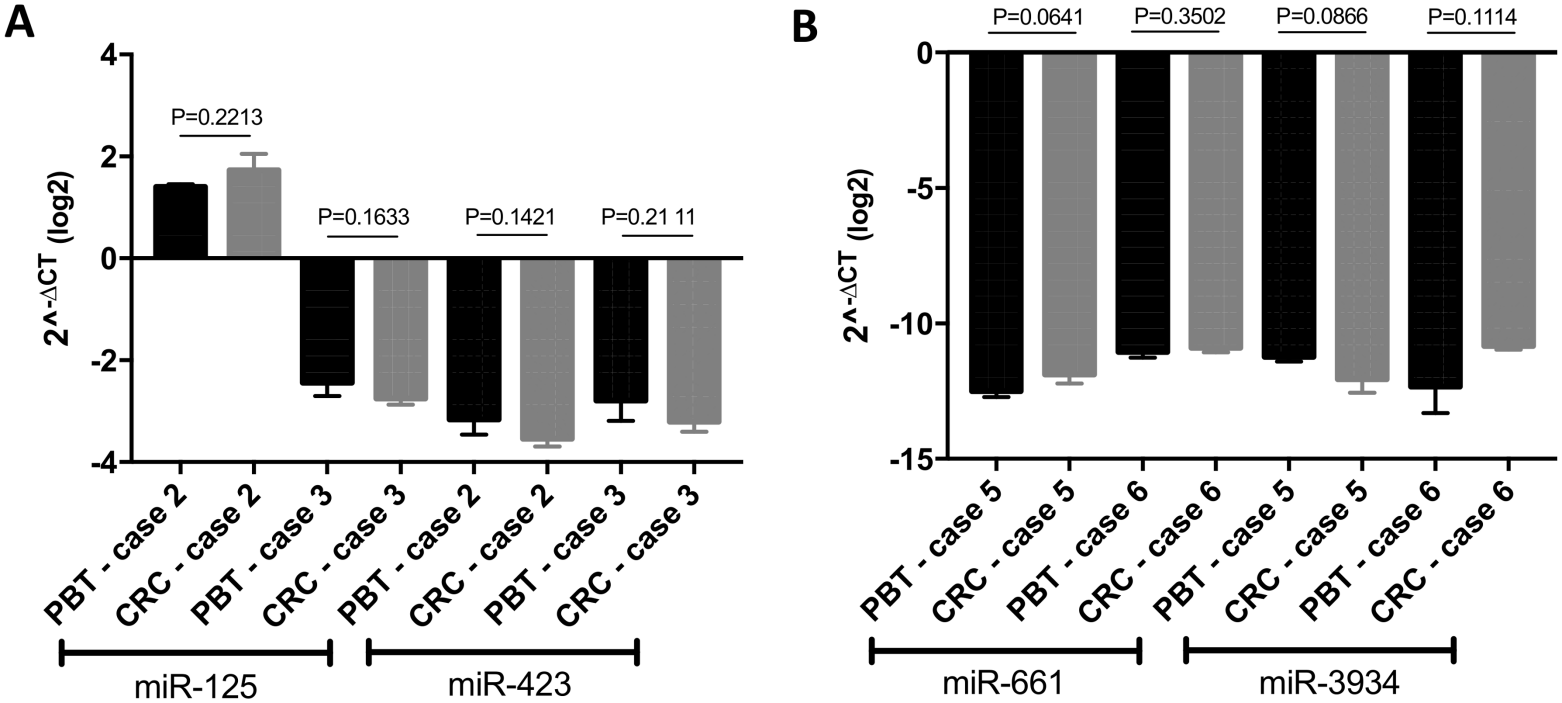
QRT-PCR analysis of a subset of paired cases of CRCs and corresponding PBTs for miRs125-5p and 423-5p (A), 661 and 3494-5p (B). No statistical difference at P value <0.05 was observed in the individual expression of these miRNAs in each of the pairs analyzed.

Individual analysis of four miRNAs (miR-125b-5p, miR-423-5p, miR-661 and miR-3934-5p), chosen alleatorily, performed by qRT-PCR in a subset of CRCs and corresponding PBTs (cases 2, 3, 5 and 6), showed no statistical difference of their expression levels among the pairs (P<0.05) (Fig 6). (The cases 1 and 4 were not included in the analysis, due to inconsistent triplicate Ct values observed in the expression analysis of their PBTs (of FFPE material) in repetitive qRT-PCR reactions).

**Fig 7:**
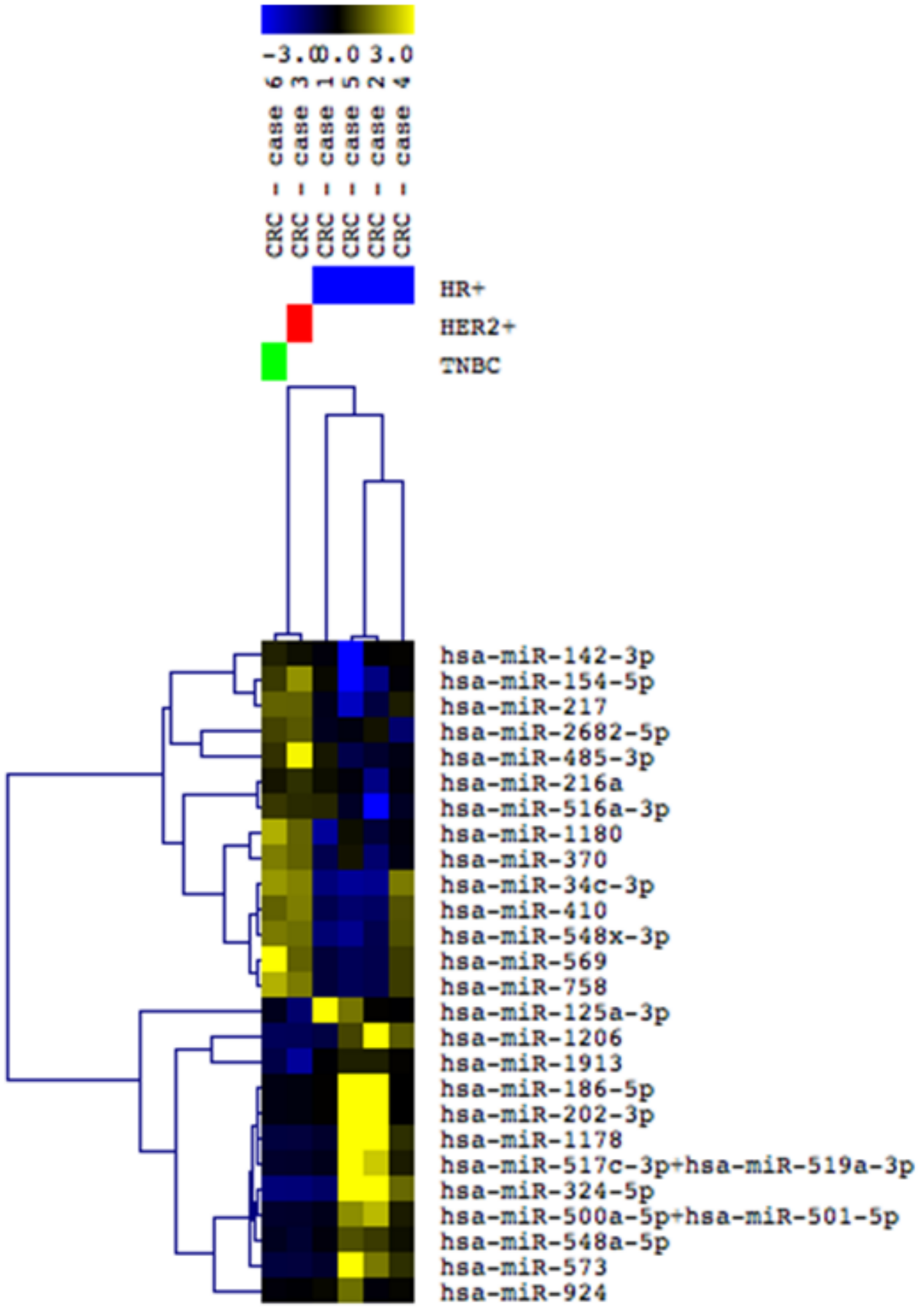
Supervised Hierarchical Clustering (SHC) analysis of the six CRCs profiled for global miRNA expression and representative of HR+/HER2- cases (cases 1, 2, 4 and 5), HER2+ (case 3) and TNBC (case 6) subtypes. Twenty-eight miRNAs were observed differentially expressed among these breast cancer subtypes (Pearson Correlation, tTest P<0.05). MiRNAs up- and down-regulated (blue) represented in yellow and blue colors, respectively.

Interestingly, based on the global miRNA profiling the CRCs clustered together according to their “IHC subtype”. A number of 28 miRNAs were observed differentially expressed among the CRCs representative of the hormone receptor (HR)+/HER2- (cases 1, 2, 4 and 5), HER2+ (case 3) and TNBC (case 6) subtypes (Pearson Correlation Analysis, t test P<0.05) (Fig 7).

## Discussion

In this study we assessed the genomic composition of six individual conditionally reprogrammed cells (CRCs) cultures directly established from the tumor tissue of six patients with invasive breast cancer. Our findings showed that the CRCs resemble and maintain the overall genomic signatures of the original primary breast tumor (PBT) from which they derived. A similar level and pattern of copy number alterations (CNAs) was observed by array-CGH in the CRCs and corresponding PBTs analyzed, with a level of overlap ranging from 72 to 100%. For the cytobands most commonly affected by CNAs, more than 95% of overlap level was observed between each CRC and their corresponding PBT. In addition, the copy number profiles of these CRCs, presented the non-random and recurrent CNAs commonly described for the intrinsic breast cancer subtypes [20–23] and DNA index of aneuploidy cells. Interestingly, case 3, the only case with positivity for HER2 protein expression, did not show amplification of the 17q21 cytoband, where this gene is located. This finding however, does not imply the absence of a focal amplification in the *HER2/NEU* gene or in genomic segments of smaller sizes that include HER2/NEU, which in this case should be verified by more specific copy number assays, such as FISH analysis [24]. In any event, although in most of the breast cancer cases overexpression of HER2 is due to gene amplification [25], other mechanisms can be involved, including aneuploidies of chromosome 17 [26,27], and/or epigenetic or posttranscriptional events [28,29]. The CRC established from the TNBC subtype (case 6) presented among the most frequent CNAs, gains at 1q21-q44, 8q24.21-q24.3, 20q11.21-q13.33 and losses at 7q11.21-q22.1 and 16p13.3-p11.1 chromosome regions, which are recurrent CNAs described in other cytogenetic studies in TNBC cases [16,30,31]. On the other hand, the CRCs of hormone receptor (HR)+/HER2- tumors, presented lower number of CNAs and less complexes array-CGH profiles (except case 2), compatible to what is reported in the intrinsic molecular luminal A breast cancer subtype [20–23]. These findings indicate the representativeness and specificity of the CNAs observed in the CRCs studied in relation to the genome of their original tumors and to the distinct molecular breast cancer subtypes.

It is relevant to point out that these analyses were performed at early CRCs’ cell passages (>P5<P10). It is of note, however, that in our previous CRC study in mouse mammary tissues we showed that the genomic (array-CGH) profiling of the CRCs resembled the ones from the non-CRC cultures at P<38 [13]. Interestingly, cytogenetic analysis of human cells immortalized by other methods of somatic reprograming, such as the ones applied for the generation of human pluripotent stem cells (hPSCs) [32–37], have shown a higher number of CNAs in early cell passages when compared to the late passage cells. These studies suggested that CNAs are either introduced during the reprogramming process or represent a sub-clone of aberrant parental cell that rapidly grows *in vitro* [32,33]. In fact, higher resolution analysis, such as whole-genome sequencing applied to hPSCs have suggested the later, considering that the CNAs observed could already be detected at low frequencies in the parental somatic cells [34,35]. Overall these studies indicate that these CNAs are effects of passages number and not of the reprogramming process *per se*. Supporting these observations, the abnormal karyotypes and CNAs that are reported in the hPSCs, occur non-randomly, affecting most commonly the chromosomes 1, 12, 17, 20 and X [32–37]. In our cases we did identify CNAs affecting some of these chromosomes, but in most cases these CNAs were also observed in the original corresponding (and uncultured) PBTs.

The targeted next generation sequencing analysis also showed that the established CRCs retained the specific gene mutations that were present in their original tumors. An analysis of three paired CRCs and PBTs (Cases 2, 4 and 6) showed that they share the same type of variants affecting the *TP53*, *FLT3*, *JAK3*, *KDR*, *PIK3CA* and *CDKN2A* genes. In the unpaired CRC (case 3) sequenced, variants in the *TP53*, *JAK3*, *KDR* and *CDKN2A* genes were observed. The same variant in the *TP53* gene that led to a codon (cCc/cGc) and aminoacid change (P72R) was observed in this CRC compared to the others CRCs and corresponding PBTs. This specific variant (COSM45985) is one of the most common polymorphisms in the *TP53* gene and was previously reported in cancer cases [38–43], although its association with cancer risk is unknown.

Missense mutations in the *JAK3*, *KDR* and *CDKN2A* genes in this CRC affected different codons and led to different aminoacid changes than the ones observed in the paired CRCs and PBTs sequenced. However, these variants were previously reported in other tumors, such as skin [44], glioblastomas [45] and leukemias [46–48] (*JAK 3*/COSM 34213), colorectal [49], prostate [50] and sarcomas [51] (*KDR*/COSM 149673). In addition, to these gene variants this CRC line presented missense mutations affecting the *MET* (COSM 1286164) and *KIT* (COSM 28026) genes, not observed in the paired cases. As for the other variants, these mutations were also reported to be present in other tumor tissues [52–54], including breast cancer [52], indicating their tumor genome representativeness.

The overall similarity of genome-wide copy number and gene mutation patterns of paired CRCs and PBTs, was also observed in our cases at the miRNA expression level. Experimental studies have shown that both the biogenesis and expression levels of miRNAs are “susceptible” to effects of cell culture conditions, including the ones that affect cellular density and contact, replicated passages and senescence [55–59]. A recent study utilized miRNA expression to compare the effects of different cell reprogramming methods in cultured cells and medium [60]. By measuring miRNA levels in the cell culture medium of pluripotent stem cells in comparison to that in cells, it was shown a constant relative abundance of miRNA level between them. Similarly, in this study we used miRNA profiling as an “epigenetic measurement” to evaluate changes in miRNA expression levels that might have occurred in the breast cancer cells cultured in the CRC system. Supervised Hierarchical Clustering (SHC) showed that the five pairs of CRCs and corresponding PBTs profiled for this analysis clustered together with high correlation coefficients, indicating the retention of the miRNA expression signature of the original tumors. QRT-PCR analysis of individual four miRNAs chosen alleatorily confirmed the similar expression of these putative miRNAs within four of the CRCs and PBTs pairs. Interestingly, despite the lower number of CRCs representative of each breast cancer “IHC subtype”, we observed that they clustered distinctly according to their “IHC subtype”. This analysis supported the suitable potential in classifying breast cancer into the molecular subtypes based in miRNA expression, as previously shown for established breast cancer cell lines and clinical cases [16,61,62].

## Conclusion

In conclusion, we demonstrated that the breast cancer CRCs evaluated in this study, maintained the overall copy number, gene mutations and miRNA expression patterns of the corresponding tumor tissue from which they derived. Considering that these CRC cultures were established from breast cancer patients with distinct clinical and histopathological characteristics, including age at onset, race, family history and hormonal status, they offer a unique and representative model of the biological breast cancer heterogeneity. Additional analyses are required to evaluate these cells at late cellular passages and to characterize them by other molecular phenotypes, to further expand their utility for cancer research.

## Supporting information

S1 Table Clinical and pathological information of the six patients’ derived breast cancer CRCs. (DOCX)

S2 Table Exome sequencing analysis of 3 pairs of CRCs and PBTs (cases 2,4 and 6) and one unpaired CRC (case 3), showing the specific gene variants observed affecting the *CDKN2A*, *FLT3*, *JAK3*, *KDR*, *KIT*, *MET*, *PIK3CA* and *TP53* genes. (DOCX)

S1 Fig DNA fingerprinting analysis of CRCs and PBTs of cases 4 and 6 for 15 short tandem repeats (STR) markers (15 autosomes and Amelogenin (X/Y). Representative traces are shown for each marker. The allele sizes are indicated under each allele (Soft Genetics, Gene Marker Software Version 1.85).
(DOCX)

S1 File Data file (miRNA raw data). (ZIP)

S2 File Data file (array-CGH raw data). (ZIP)

S3 File Data file (array-CGH raw data). (ZIP)
