# Supplementary material for: Genomic comparison of early-passage conditionally reprogrammed breast cancer cells to their corresponding primary tumors
Source: PLoS One. 2017 Oct 19;12(10):e0186190. doi: 10.1371/journal.pone.0186190 (PMC5648156; doi:10.1371/journal.pone.0186190)
Supplement: S2 Table — (DOCX) [file pone.0186190.s002.docx]

**Supplementary Information**

**S2 Table.** Targeted next generation sequencing analysis of 3 pairs of CRCs and PBTs (cases 2, 4 and 6) and one unpaired CRC (case 3), showing the specific gene variants observed affecting the *CDKN2A, FLT3, JAK3, KDR, KIT, MET, PIK3CA* and *TP53* genes.

S2 Table

|  | | Sample | | Gene | Chromosome | | Genomic Coordinate | | Reference Allele | | Variant Allele | Alt Variant Freq | | Read Depth | Allelic Depths |
| --- | --- | --- | --- | --- | --- | --- | --- | --- | --- | --- | --- | --- | --- | --- | --- |
| case 2 | CRC-case 2 | | FLT3 | | 13 | 28610183 | | A | | G | | 0.995 | 403 | | 2401 |
|  | PBT-case 2 | | FLT3 | | 13 | 28610183 | | A | | G | | 0.99 | 299 | | 2297 |
|  | CRC-case 2 | | TP53 | | 17 | 7579472 | | G | | C | | 1 | 525 | | 0,525 |
|  | PBT-case 2 | | TP53 | | 17 | 7579472 | | G | | C | | 0.99 | 170 | | 2168 |
|  |  | |  | |  |  | |  | |  | |  |  | |  |
| case 4 | CRC-case 4 | | FLT3 | | 13 | 28610183 | | A | | G | | 1 | 387 | | 2385 |
|  | PBT-case 4 | | FLT3 | | 13 | 28610183 | | A | | G | | 0.99 | 270 | | 2268 |
|  | CRC-case 4 | | TP53 | | 17 | 7579472 | | G | | C | | 0.54 | 387 | | 176207 |
|  | PBT-case 4 | | TP53 | | 17 | 7579472 | | G | | C | | 0.36 | 193 | | 124,69 |
|  | CRC-case 4 | | KDR | | 4 | 55972974 | | T | | A | | 0.99 | 461 | | 4457 |
|  | PBT-case 4 | | KDR | | 4 | 55972974 | | T | | A | | 0.98 | 352 | | 8343 |
|  |  | |  | |  |  | |  | |  | |  |  | |  |
| case 6 | CRC-case 6 | | TP53 | | 17 | 7579472 | | G | | C | | 1 | 511 | | 0,511 |
|  | PBT-case 6 | | TP53 | | 17 | 7579472 | | G | | C | | 1 | 9 | | 0,9 |
|  | CRC-case 6 | | CDKN2A | | 9 | 21970979 | | C | | A | | 0.54 | 412 | | 191221 |
|  | PBT-case 6 | | CDKN2A | | 9 | 21970979 | | C | | A | | 0.4 | 15 | | 9,6 |
|  | CRC-case 6 | | JAK3 | | 19 | 17954215 | | G | | T | | 0.47 | 340 | | 178160 |
|  | PBT-case 6 | | JAK3 | | 19 | 17954215 | | G | | T | | 0.82 | 11 | | 2,9 |
|  | CRC-case 6 | | PIK3CA | | 3 | 178927410 | | A | | G | | 0.44 | 803 | | 449,353 |
|  | PBT-case 6 | | PIK3CA | | 3 | 178927410 | | A | | G | | 0.37 | 41 | | 26,15 |
|  |  | |  | |  |  | |  | |  | |  |  | |  |
| case 3 | CRC-case 3 | | MET | | 7 | 116340214 | | G | | A | | 0.46 | 346 | | 188158 |
|  | CRC-case 3 | | CDKN2A | | 9 | 21970916 | | C | | T | | 0.34 | 140 | | 93,47 |
|  | CRC-case 3 | | JAK3 | | 19 | 17945696 | | C | | T | | 0.5 | 218 | | 110108 |
|  | CRC-case 3 | | KIT | | 4 | 55593464 | | A | | C | | 0.52 | 262 | | 125137 |
|  | CRC-case 3 | | KDR | | 4 | 55979558 | | C | | T | | 0.42 | 229 | | 134,95 |
|  | CRC-case 3 | | TP53 | | 17 | 7579472 | | G | | C | | 1 | 197 | | 1196 |

Cont. S2 Table

| Mutation Type | Codon Change | Amino Acid Change | COSMIC ID | dbSNP ID | FATHMM prediction | SIFT |
| --- | --- | --- | --- | --- | --- | --- |
| splice region | . | . | . | rs2491231 | . | . |
| splice region | . | . | . | rs2491231 | . | . |
| missense | cCc/cGc | P72R | COSM45985 | rs1042522 | Neutral (score 0.22) | tolerated(0.56) |
| missense | cCc/cGc | P72R | COSM45985 | rs1042522 | Neutral (score 0.22) | tolerated(0.56) |
|  |  |  |  |  |  |  |
| splice region | . | . | . | rs2491231 | . | . |
| splice region | . | . | . | rs2491231 | . | . |
| missense | cCc/cGc | P72R | COSM45985 | rs1042522 | Neutral (score 0.22) | tolerated(0.56) |
| missense | cCc/cGc | P72R | COSM45985 | rs1042522 | Neutral (score 0.22) | tolerated(0.56) |
| missense | caA/caT | Q472H | COSM149673 | rs1870377 | Neutral (score 0.07) | tolerated(0.1) |
| missense | caA/caT | Q472H | COSM149673 | rs1870377 | Neutral (score 0.07) | tolerated(0.1) |
|  |  |  |  |  |  |  |
| missense | cCc/cGc | P72R | COSM45985 | rs1042522 | Neutral (score 0.22) | tolerated(0.56) |
| missense | cCc/cGc | P72R | COSM45985 | rs1042522 | Neutral (score 0.22) | tolerated(0.56) |
| missense | Gca/Tca | A127S | COSM12514 | rs6413464 | Neutral (score 0.36) | deleterious(0.03) |
| missense | Gca/Tca | A127S | COSM12514 | rs6413464 | Neutral (score 0.36) | deleterious(0.03) |
| missense | Cca/Aca | P132T | COSM34216 | rs3212723 | Pathogenic (score 0.90) | tolerated(0.39) |
| missense | Cca/Aca | P132T | COSM34216 | rs3212723 | Pathogenic (score 0.90) | tolerated(0.39) |
| missense | atA/atG | I391M | COSM328028 | rs2230461 | none (score 0.56) | tolerated(0.33) |
| missense | atA/atG | I391M | COSM328028 | rs2230461 | none (score 0.56) | tolerated(0.33) |
|  |  |  |  |  |  |  |
| missense | cGa/cAa | R359Q | COSM1286164 | rs201274041 | Pathogenic (score 0.87) | tolerated(0.23) |
| missense | Gcg/Acg | A148T | . | rs3731249 | . | deleterious(0.01) |
| missense | Gtc/Atc | V722I | COSM34213 | rs3213409 | Neutral (score 0.23) | deleterious(0.04) |
| missense | Atg/Ctg | M541L | COSM28026 | rs3822214 | Pathogenic (score 0.74) | tolerated(0.34) |
| missense | Gta/Ata | V297I | COSM1131107 | rs2305948 | Pathogenic (score 0.97) | deleterious(0.05) |
| missense | cCc/cGc | P72R | COSM45985 | rs1042522 | Neutral (score 0.22) | tolerated(0.56) |
