# Supplementary material for: Genomic comparison of early-passage conditionally reprogrammed breast cancer cells to their corresponding primary tumors
Source: PLoS One. 2017 Oct 19;12(10):e0186190. doi: 10.1371/journal.pone.0186190 (PMC5648156; doi:10.1371/journal.pone.0186190)
Supplement: S1 Fig — Representative traces are shown for each marker. The allele sizes are indicated under each allele (Soft Genetics, Gene Marker Software Version 1.85). (DOCX) [file pone.0186190.s003.docx]

**Supplementary Information**

**S1 Fig.** DNA fingerprinting analysis of CRCs and PBTs of cases 4 and 6 for short tandem repeats (STR) markers (15 autosomes and Amelogenin (X/Y). Representative traces are shown for each marker. The allele sizes are indicated under each allele (Soft Genetics, Gene Marker Software Version 1.85).


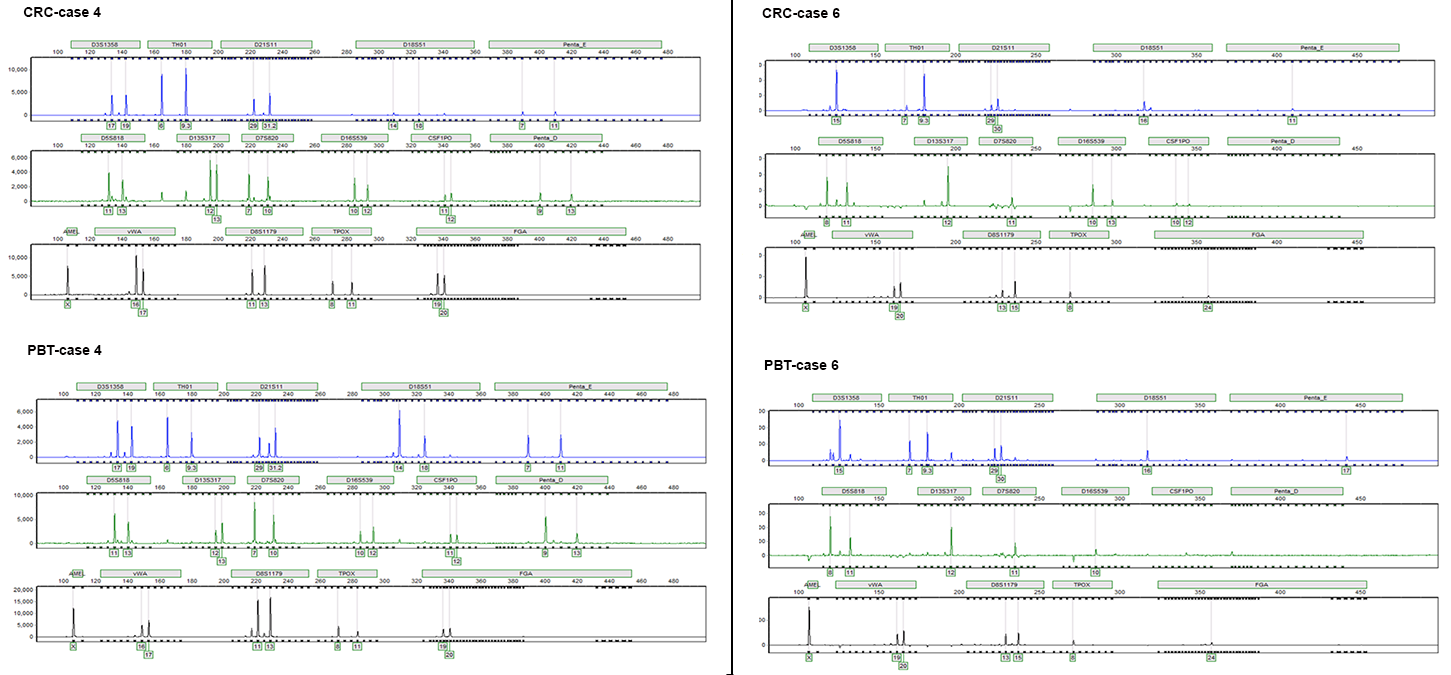


S1 Fig.
